# Role of Berry Anthocyanins and Phenolic Acids on Cell Migration and Angiogenesis: An Updated Overview

**DOI:** 10.3390/nu11051075

**Published:** 2019-05-15

**Authors:** Panagiotis Tsakiroglou, Natalie E. VandenAkker, Cristian Del Bo’, Patrizia Riso, Dorothy Klimis-Zacas

**Affiliations:** 1 School of Food and Agriculture, University of Maine, Orono, ME 04469, USA; panagiotis.tsakiroglou@maine.edu (P.T.); natalie.marchi@maine.edu (N.E.V.); 2 Department of Food, Environmental and Nutritional Sciences (DeFENS), Università degli Studi di Milano, 20123 Milan, Italy; cristian.delbo@unimi.it (C.D.B.); patrizia.riso@unimi.it (P.R.)

**Keywords:** cell migration, angiogenesis, anthocyanins, phenolic acids, berries, extracts, polyphenols, HUVECs

## Abstract

Cell migration is a critical process that is highly involved with normal and pathological conditions such as angiogenesis and wound healing. Important members of the RHO GTPase family are capable of controlling cytoskeleton conformation and altering motility characteristics of cells. There is a well-known relationship between small GTPases and the PI3K/AKT pathway. Endothelial cell migration can lead to angiogenesis, which is highly linked to wound healing processes. Phenolics, flavonoids, and anthocyanins are major groups of phytochemicals and are abundant in many natural products. Their antioxidant, antimicrobial, anti-inflammatory, antidiabetic, angiogenenic, neuroprotective, hepatoprotective, and cardioprotective properties have been extensively documented. This comprehensive review focuses on the in vitro and in vivo role of berry extracts and single anthocyanin and phenolic acid compounds on cell migration and angiogenesis. We aim to summarize the most recent published studies focusing on the experimental model, type of berry extract, source, dose/concentration and overall effect(s) of berry extracts, anthocyanins, and phenolic acids on the above processes.

## 1. Introduction

### 1.1. Cell Migration

One fundamental process common to cell morphogenesis, immune function, regeneration, and disease is cell migration [1,2]. Cellular crosstalk exists during migration which allows for collective cell movement in the same direction and at a similar speed [1]. Chemotaxis, haptotaxis, and mechanotaxis are the three major mechanisms that are utilized by endothelial cells during migration and angiogenesis [2,3]. Growth factors, i.e., vascular endothelial growth factor (VEGF), cytokines, and high blood glucose, induce cell migration [2]. Additionally, the production of NO from eNOS, activated by AKT/PKB plays a significant role in endothelial cell migration [3].

Early studies demonstrate the importance of small G proteins on cell motility [4,5,6,7]. During cell migration, the cell establishes a front-to-rear polarity axis involving small GTPases including RHOA, RAC and CDC42, members of the Rho family and RAC is involved in the formation of lamellipodia at the leading edge of migrating cells [4]. At the rear, RHOA promotes actin–myosin contraction and is required during focal adhesions while CDC42 is not directly involved in cell migration/movement but is essential for cell polarity that controls the direction of cell movement [4].

Promotion of cell migration is crucial in processes such as wound healing and tissue regeneration/renewal associated with burns, diabetes mellitus, ischemic conditions, and aging. However, in many chronic diseases such as atherosclerosis, tumor growth, and various fibrotic conditions, excessive cell migration results in enhanced invasion of cells across an extracellular matrix [3].

### 1.2. Angiogenesis

In cardiovascular biology there are three different types of blood vessel formation (angiogenesis, arteriogenesis, and vasculogenesis) [8]. The formation of blood vessels from pre-existing ones is known as angiogenesis, a highly complex and coordinated process [9,10]. Arteriogenesis is the de novo formation of blood vessels [11,12]; vasculogenesis is the in situ formation of blood vessels from vascular progenitor cells and circulating endothelial progenitor cells (EPCs) [13,14]. All three processes are subcategories of neovascularization that can occur in adults when there is a state of ischemia [8]. Angiogenesis is a normal biological process that starts in the early stages of biological development [15]. Normally angiogenesis is active after birth, and in adulthood it occurs during the ovarian cycle, in pregnancy [15], and during wound healing and repair [16]. A member of the AGC kinases, AKT kinase, plays a key role in angiogenesis [17]. Upstream regulators of AKT in the cardiovascular system are platelet derived growth factor (PDGF), vascular endothelial growth factor (VEGF), epithelial growth factor (EGF), and basic fibroblast growth factor (bFGF) [17,18,19]. Secretion of VEGF can be stimulated by AKT through the AKT-PI3K pathway [20,21,22] and AKT can directly phosphorylate eNOS which plays a major role in angiogenesis and vascular permeability [23].

Control of angiogenesis can be a therapeutic tool during chronic diseases (cardiovascular disease, tumor growth, diabetic angiopathy, ischemic conditions), wound healing, tissue regeneration and remodeling, where the balance of blood vessel formation is controlled by pro-and anti-angiogenic factors [24]. Even though a plethora of angiogenic factors have been reported, the primary pathway that modulates angiogenesis is initiated mainly from hypoxia-inducible factor (HIF)-1α expression [8]. Nearly four decades ago, it was hypothesized that inhibition of angiogenesis could be a modality to treat human cancer effectively [25]. Clinical trials have documented promising results that inhibition of angiogenesis can be an important target for cancer and other diseases [25]. Therapeutic promotion of angiogenesis can also play a significant role in diseases such as ischemic disorders [26], tissue remodeling [27], and wound healing [28,29,30]. Chronic diseases such as diabetes mellitus lead to impaired wound healing, a result of unbalanced angiogenesis due to ischemia in the extremities [28,29,30]. A better understanding of molecular pathways involved in angiogenesis is critical [25]. 

Recent evidence documents the important pro- and/or antiangiogenic role of phytochemicals (polyphenols) on angiogenesis [31] and their role in chronic diseases [32,33,34,35]. Studies have focused on the HIF autoregulated vascular growth factor (VEGF) gene expression as well as the targeted therapeutic delivery of phytochemical compounds to tissues [36]. 

### 1.3. Phytochemicals

Epidemiologic, clinical, and experimental studies have documented that the consumption of nutraceutical products have health-promoting effects. Bioactive compounds (BACs) are essential and non-essential nutrients that naturally occur in nature, particularly in plants and plant products [37]. Phytochemicals are identified as plant products that are non-essential BAC. These compounds are secondary metabolites that play an essential role in the plant’s reproduction, growth, protective mechanisms, odor, taste, and color [38]. The plant’s composition of these phytochemicals differs depending on the plant type, light intensity, maturity, storage, climate, and soil conditions. Collectively there are more than 10,000 identified compounds but still a large percentage remains unknown [39]. 

Major classes of these chemical structures are carotenoids, phenolics, alkaloids, nitrogen-containing and organosulfur compounds [39]. Polyphenols are the largest group of phytochemicals and are classified by the arrangement of the carbon atoms and their substituents [40]. They contain a benzene ring, a carboxylic group, and one or more hydroxyl and/or methoxyl group [41] and are divided into two major groups; flavonoids and non-flavonoids [40,42]. They are further divided into 16 classes based on their carbon chain [41]. Non-flavonoids consist of phenolic acids, stilbenes, coumarins, and tannins. Flavonoids consist of a variety of classes including flavonols, flavones, flavanols, flavanones, isoflavonoids, and anthocyanins [43]. 

Flavonoids are the most studied group of polyphenolic compounds, that are characterized by their benzo-γ-prone structure [44]. They are naturally found in teas, fruits, vegetables, seeds, and nuts, with small amounts in red wine, coffee, and cocoa [45]. Flavonols have a 3-hydroxyflavone skeleton found most abundant in onions, leafy vegetables, broccoli, and berries [46]. Flavones have a 2-phenylchromen-4-one backbone, found in cereals and herbs [47]. The richest source of flavanols is cocoa and tea, with a 2-phenyl-3, 4-dihydro-2 H-chromen-3-ol skeleton [48]. Flavanones are highly present in citrus plants [49]. Isoflavonoids possess a 3-phenylchroman backbone, with the highest content found in soya and legumes [50]. 

Anthocyanins are the glycosylated version of flavylium (2-phenylchromenylium), which is found more frequently in the 3-OH position [51]. They are the largest group of water-soluble molecules contributing to the bright pigment of berry fruit [52]. Scientists have reported over 500 different anthocyanins isolated from plant products [51]. The most abundant anthocyanins are: pelargonidin (Pg-3-glc), cyanidin (Cy-3-glc), peonidin (Pn-3-glc), delphinidin (Dp-3-glc), petunidin (Pt-3-glc), and malvidin (Mv-3-glc) [51,53,54]. Anthocyanins are highly reactive molecules and are very sensitive to many factors such as light, temperature, oxygen, enzymes, and pH [51]. 

An individual on average can consume 180–215 mg/day of anthocyanins [55,56]. However, anthocyanins are not highly bioavailable resulting in low concentrations of native forms in the blood and they are excreted in the urine within 2–8 h [52,57,58]. Absorption of anthocyanins and other flavonoids takes place rapidly in the stomach and the small intestine [53,59]. Bilitranslocase is an enzyme involved in this process [59] and acts as a flavonoid membrane transporter [60]. After entering the circulatory system, they pass through the liver before reaching target organs [53]. Anthocyanins that are not absorbed within 15 min–2 h, are exposed to gut microbiota mainly in the colon [53]. 

Phenolic acids are a subcategory of phenolics that are divided in two major groups, hydroxybenzoic acids and hydroxycinnamic acids. Phenolic acids are characterized by hydroxylated aromatic rings [61]. Hydroxybenzoic acids are mainly found in complex structures such as lignin, while hydroxycinnamic acids are found in cell wall structure components [62]. Limited information is available regarding phenolic acid metabolism in the human body after consumption [63]. It is suggested that phenolic acids are metabolized quickly after consumption and undergo conjugation reactions after absorption from the gastrointestinal tract [62]. These conjugate forms have been documented to alter the bioavailability of the initial structure [62]. 

Berry fruits contain an abundance of anthocyanin and phenolic acid compounds. Due to their favorable aroma and taste, they are common in the human diet. In the United States, commonly consumed berries are blackberry, black raspberry, blueberry, cranberry, red raspberry, and strawberry, each with a unique polyphenolic profile [52]. 

An abundance of research demonstrates that polyphenols have a wide range of biological effects, including antioxidant, antimicrobial, anti-inflammatory, antidiabetic, angiogenenic, neuroprotective, hepatoprotective, and cardioprotective properties [42,63,64,65,66,67]. These compounds individually or in combination may be responsible for their protective effects such as delaying the aging process and reducing the risk of chronic diseases and cancers [68]. As mentioned earlier, their beneficial effects and targeted tissue delivery is of great interest for further investigation associated with their pro- or anti-angiogenic potential [31]. 

In this review, we focused on the in vitro and in vivo studies using berry extracts and single anthocyanin and phenolic acid compounds and their role on cell migration and angiogenesis (Table 1 and Table 2). PubMed (www.ncbi.nlm.nih.gov/pubmed) and Web of Science (www.webofknowledge.com) databases were screened for studies published in English between 2000–January 2019 excluding cancer cell models and applications. Various combinations of several key terms were used: (phytochemical OR polyphenols OR anthocyanins OR phenolic acids OR fruit OR berries OR berry extracts) AND (angiogenesis OR neovascularization OR arteriogenesis OR cell migration). 

## 2. Endothelial Cell Migration and Angiogenesis: Role of Anthocyanins

Table 1 presents the role of berry anthocyanin extracts or single compounds on cell migration and angiogenesis in vivo and in vitro. Martin et al. [69], exposed bovine aortic endothelial cells (BAECs) with commercially obtained delphinidin (10^−2^ g/L) and demonstrated that delphinidin exerted antiproliferative characteristics. Similarly, Lamy et al. [70] studied the effects of commercially obtained delphinidin (25 μM) on HUVECs and documented inhibition of vessel (tube) formation, antiangiogenic activity, and inhibition of VEGF-induced migration [70]. Furthermore, HUVEC and pulmonary aortic smooth muscle cells (PASMCs) treated with commercially obtained delphinidin (25 μM), inhibited activation of the platelet-derived growth factor receptor β [71]. In other studies, HUVECs were exposed to 10^−2^ g/L delphinidin in the presence or absence of proangiogenic factor VEGF [72]. In the presence of VEGF, delphinidin-treated HUVECs were unable to promote mitochondrial biogenesis and activate the Akt pathway [72]. 

In 2010, Matsunaga et al. [73] treated HUVECs with commercially obtained delphinidin, cyanidin, and malvidin (0.3, 1, 3, and 10 μM). Exposure of HUVECs to all three compounds individually, resulted in an equal inhibition of VEGF-induced angiogenesis [73]. In other experiments, HUVEC and human dermal microvascular endothelial cells (HMVECs) were treated with 5, 10, 15, 10, or 25 μM of each apigenin, delphinidin, ellagic acid or luteolin respectively [74]. Each compound inhibited cell migration in the presence of IL-6. The role of apigenin and luteolin on proliferation and migration may be mediated through JAK/STAT3 and MAPK signaling pathways [74]. Commercially obtained quercetin (0.1, 1, 10, 25, and 50 μM/L) treated HUVECs, suppressed inflammatory angiogenesis via the attenuation of stimulated COX-2 expression, prostanoid production, and MMP-9 release [75]. Similarly, quercetin (50, 100, and 200 μM) inhibited the development of intersegmental vessel, dorsa aorta, and posterior cardinal vein in a transgenic zebrafish model [76]. In addition, quercetin effectively inhibited HUVEC migration, proliferation, and tube formation [76]. 

Son et al. [77] treated aortic smooth muscle cells (HASMCs) and HUVECs with commercially available pelargonidin and pelargonidin-3-glucoside (10, 20, and 40 μM) and demonstrated that pelargonidin but not its glucoside conjugated form, pelargonidin-3-glucoside, exhibited strong inhibitory effects of HASMC proliferation and migration. Further investigation with an aortic ring-sprouting assay, documented that pelargonidin significantly reduced PDGF-BB-induced aortic sprouting [77]. However, pelargonidin did not have inhibitory effects on the proliferation and migration of HUVECs [77]. A similar study treated HASMCs with individual commercial compounds, petunidin, delphinidin, cyanidin, pelargonidin, malvidin, and peonidin [78]. The six anthocyanins demonstrated antiangiogenetic characteristics with petunidin exhibiting the strongest inhibitory effect on HASMCs during cell migration [78]. In general, single anthocyanins potentially inhibit cell migration and angiogenesis in HUVEC and MUVEC cell culture models at concentrations from 0.1–200 μM, depending on cell type.

An ex vivo study evaluated the effects of the anthocyanin cyanidin-3-O-b-glucoside commercial compound on cell adhesion, cell migration, and tube formation [79]. Zhang et al. [79] isolated endothelial progenitor cells (EPCs) from blood and bone marrow of both nondiabetic and diabetic apoE^-/-^ mice. The diabetic mice were fed the AIN-93 diet, or an AIN-93 diet supplemented with cyanidin-3-O-b-glucoside (0.2 % (wt/wt)) for six weeks. Cyanidin-3-O-b-glucoside improved cell adhesion and cell migration indicating beneficial effects of anthocyanins. Moreover, tube formation was normalized compared to the diabetic mice without the cyanidin-3-O-b-glucoside supplementation [79]. 

Treatment with both crude extracts (0.075% (w/v)) of black raspberries inhibited angiogenic initiation and vessel growth in the human placental vein angiogenesis model (HPVAM) [80]. Anthocyanin extracts (0.3, 1, 3, 10, and 30 μM) from bilberry inhibited angiogenesis both in vitro (HUVECs) and in vivo (C57BL/6 mice) by the reduction in cell proliferation and migration through inhibition of p-ERK 1/2 and p-Akt [81]. Similarly, dietary supplementation of a bilberry anthocyanin-rich extract (0.02%) attenuated atherosclerotic lesion development in apolipoprotein E-deficient (apoE^-/-^) mice [82]. After a two-week treatment period, nutrigenomic analysis identified that 1261 genes were modulated by the bilberry extract in the isolated aorta. Bioinformatic analysis revealed that these genes are implicated in different cellular processes such as oxidative stress, inflammation, trans-endothelial migration, and angiogenesis processes associated with atherosclerosis development/protection [82]. 

Five rabbit-eye blueberry varieties (Centurion, Maru, Rahi, Ono, Tifblue) were used to extract anthocyanin and chlorogenic acid [83]. The anthocyanin extracts demonstrated anti-angiogenic properties in the Chicken Chorioallantoic Membrane (CAM) assay [83]. Bae et al. [84] investigated the in vitro and in vivo anti-angiogenetic activity of ethanol extracts of Korean crowberry. Extract concentrations of 31.3, 62.5, 125, 250, and 500 μg/mL used inhibited angiogenesis in HUVECs by hindering tube formation and cell proliferation. Additionally, the ethanol extracts of Korean crowberry suppressed angiogenetic activity in vivo using the CAM assay [84]. 

Recently, Tsakiroglou et al. [85] exposed HUVECs to an anthocyanin fraction extracted from wild blueberry powder with the following concentrations 0.002, 8, 15, 60, and 120 μg/mL. The improved wound healing assay was used to evaluate the effect of anthocyanins on endothelial cell migration. Anthocyanins at 60 μg/mL were found to inhibit endothelial cell migration affecting not only the speed of migrating cells but also expression levels of critical gene encoding molecules important for cell movement and cytoskeletal arrangement and adhering junctions [85].

Thus, single anthocyanins as well as anthocyanin-rich fractions from several berry extracts potentially inhibit cell migration and angiogenesis in vitro, in vivo, and ex vivo, having implications for diseases that benefit from inhibition of cell migration and angiogenesis. 

## 3. Endothelial Cell Migration and Angiogenesis: Role of Phenolic Acids

Scant and conflicting evidence exists on the role of phenolic acids on cell migration and angiogenesis. Studies conducted with individual phenolic acids as well as berry extracts are presented below. 

An in vitro study treated HUVECs with chlorogenic acid (10 μM) [86]. Chlorogenic acid significantly inhibited hypoxia-induced network formation by inducing endothelial cells to form shorter and broken tubes and reduced mobility of hypoxia-induced HUVECs. Additionally, chlorogenic acid was able to significantly inhibit cell invasion due to hypoxic conditions [86]. However, commercially obtained ferulic acid (10^−6^–10^−4^ M), classified under the same polyphenol family as chlorogenic acid, induced cell migration and tube formation of HUVECs [87] by upregulating VEGF and PDGF pathways. In other experiments (ferulic acid 10^−6^–10^−5^ M) resulted in increased percentage of neovascularization utilizing the CAM assay [87]. Commercially obtained p-coumaric acid (0.5, and 1.0 mM) though, suppressed sprouting of endothelial cells in rat aortic rings and inhibited tube formation and migration of ECV304 cells [88]. 

Total phenolics were extracted from red raspberries and the extracts at various concentrations 10, 25, 50, or 100 μg GAE/mL were introduced to HMVECs. Results documented that the total phenolic extract (GI_50_ = 87.64 ± 6.59 GAE/mL) of red raspberries reduced cell viability and proliferation in a dose-dependent manner [89]. 

Chlorogenic acid extracts from five rabbit-eye blueberry varieties (30 μL from 180 mL crude extract) correlated with antioxidant activity but not with anti-angiogenesis properties [83]. The same study used anthocyanin extracts that resulted in anti-angiogenetic characteristics, suggesting that angiogenic activity depends on the type of phytochemical and not on total antioxidant activity. 

A recent study, utilizing an in vitro wound healing assay documented increased speed of endothelial cell migration when HUVECs were treated with a phenolic acid extract from wild blueberries, primarily containing chlorogenic acid at specific concentrations (0.002, 8, 15, 60, and 120 μg/mL). Concentrations of 0.002, 60, and 120 μg/mL documented to significantly increase endothelial cell migration compared to untreated cells. Moreover, the phenolic acid fraction increased markers (RHOA and RAC1) critical for cell migration and cytoskeletal reformation not only at gene level but at protein level as well [85].

Thus, the above studies have observed both anti- and proangiogenic properties of phenolic acids, either as individual compounds or as extracts with the possible involvement of VEGF, PDGF, and HIF-1a signaling pathways. 

## 4. Conclusions

We conclude that the majority of studies document the anti-angiogenic role of anthocyanins, but results are mixed on the role of phenolic acids on cell migration and angiogenesis and the signaling pathways they modulate as they relate to chronic disease. The contradicting results may be attributed to the use of different types of phenolics and combinations, concentration ranges, their respective metabolites, and use of extracts versus single compounds. Additional factors may be the use of single cell types or co-cultures, the length of exposure and sampling at different exposure times, stimulated or unstimulated cell cultures, and type of stimulation. For in vivo studies, the type of animal model used and the disease state targeted may also contribute to the conflicting results. This calls for scientific collaboration in the use of common extracts and/or bioactive compounds as well as similar experimental design and protocols to make valid conclusions. Future studies should also target signaling pathways and molecules associated with cell migration and angiogenesis to reveal the mechanisms by which the above phytochemicals confer their differential action. Additionally, the potential and efficacy of berry bioactive compounds for targeted cell delivery associated with chronic diseases and health conditions should be explored. 

## Figures and Tables

**Table 1:** Role of anthocyanins on cell migration and angiogenesis.

Reference	Model	Fraction(s)	Source	Concentration	Overall Effect
Martin, S., (2003) [69]	BAECS	Delphinidin chloride	Commercially obtained	10^−2^ g/L	Anti-angiogenesis
Lamy, S., et al. (2006) [70]	HUVECs	Delphinidin	Commercially obtained	25 μM	Anti-angiogenesis
Lamy, S. et al. (2008) [71]	HUVECs PASMCs	Cyanidin, delphinidin, pelargonidin, and petunidin	Commercially obtained	25 μM	Anti-angiogenesis
Duluc, L., et al. (2014) [72]	HUVECs	Delphinidin	Commercially obtained	10^−2^ g/L	Anti-angiogenesis
Matsunaga, N., et al. (2010) [73]	HUVECs	Delphinidin, cyanidin, and malvidin	Commercially obtained	0.3, 1, 3 and 10 μM	Anti-angiogenesis
Lamy, S., et al. (2012) [74]	HUVECs HMVECs	Apigenin, delphinidin, ellagic acid, and luteolin	Commercially obtained	5, 10, 15, 20 and 25 μM	Anti-angiogenesis
Scodittie, E., et al. (2012) [75]	HUVEC	Quercetin	Commercially obtained	0.1, 1, 10, 25 and 50 μmol/L	Anti-angiogenesis
Zhao, D., et al. (2014) [76]	HUVEC Transgenic zebrafish embryos	Quercetin	Commercially obtained	50, 100 and 200 μM	Anti-angiogenesis
Son, J., et al. (2014) [77]	HASMCs HUVECs	Pelargonidin chloride and pelargonidin-3-glucoside chloride	Commercially obtained	10, 20 and 40 μM	Anti-angiogenesis
Son, J., et al. (2014) [78]	HASMCs Sprague-Dawley Rats	Petunidin, Delphinidin, Cyanidin, Pelargonidin, Malvidin, and Peonidn	Commercially obtained	2.5, 5, 10, 20 and 40 μM	Anti-angiogenesis
Zhang, Y., et al. (2013) [79]	apoE^-/-^ Mouse Model	Cyanidin-3-O-b-glucoside (C3G)	Commercially obtained	0.2% (w/w)	Pro-angiogenesis
Liu, Z., et al. (2005) [80]	HPVAM	Crude Extract	Frozen whole black raspberries	0.075% (w/v)	Anti-angiogenesis
Matsunaga, N., et al. (2010) [81]	C57BL/6 Mice HUVECs	Anthocyanins	Bilberry	0.3, 1, 3, 10 and 30 μM	Anti-angiogenesis
Mauray, A., et al. (2012) [82]	apoE-/- Mouse Model	Anthocyanins	Bilberry	Diet supplemented with 0.02% of Bilberry	Anti-angiogenesis
Vuthijumnonk, J., et al. (2015) [83]	CAM	Anthocyanins	Rabbit-eye Blueberry	30 μL from 180 mL crude extract	Anti-angiogenesis
Bae, K., et al. (2016) [84]	HUVEC CAM	Ethanol Extract	Crowberry	31.3, 62.5, 125, 250 and 500 μg/mL 25, 50, 100 and 200 μg	Anti-angiogenesis
Tsakiroglou, P. et al., (2019) [85]	HUVEC Ibidi wound healing assay	Anthocyanin extract	Wild blueberry	0.002, 8, 15, 60 and 120 μg/mL	Anti-angiogenesis

BAECs: Bovine aortic endothelial cells, HUVECs: Human umbilical vein endothelial cells; PASMCs: pulmonary aortic smooth muscle cells; HMVECs: Human microvascular endothelial cells; HASMCs: Human aortic smooth muscle cells; HPVAM: Human placental vein angiogenesis model; CAM: Chick Chorioallantoic Membrane.

**Table 2:** Role of phenolic acids on cell migration and angiogenesis.

Reference	Model	Fraction(s)	Source	Concentration	Overall Effect
Park, J., et al. (2015) [86]	HUVECs	Chlorogenic Acid	Commercially obtained	2 and 10 μM	Anti-angiogenesis
Lin, C.M., et al. (2010) [87]	HUVECs CAM	Ferulic acid	Commercially obtained	(10^−6^–10^−4^ M) (10^−6^–10^−5^ M)	Pro-angiogenesis
Kong, C., et al. (2013) [88]	ECV304 cells Sprague-Dawley Rats	*p*-Coumaric Acid	Commercially obtained	0.5 and 1 mM	Anti-angiogenesis
Sousa, M., et al. (2016) [89]	HMVECs	Total Phenolic Extract	Red Raspberries	10, 25, 50 and 100 μg GAE/mL	Anti-angiogenesis
Vuthijumnonk, J., et al. (2015) [83]	CAM	Chlorogenic Acid	Rabbit-eye Blueberry	30 μL from 180 mL crude extract	Pro-angiogenesis
Tsakiroglou, P., et al., (2019) [85]	HUVEC Ibidi wound healing assay	Phenolic acid fraction	Wild blueberry	0.002, 8, 15, 60 and 120 μg/mL	Pro-angiogenesis

HUVECs: Human umbilical vein endothelial cells; HMVECs: Human microvascular endothelial cells; CAM: Chick Chorioallantoic Membrane.
